# Forest Structure, Diversity, and Regeneration in a Community‐Managed Forest of Nepal: A Model for Carbon Sequestration and Sustainable Management

**DOI:** 10.1002/pei3.70044

**Published:** 2025-03-16

**Authors:** Tika Ram Poudel, Prakash Chandra Aryal, Muhammad Tayyab Khan, Nathan James Roberts, Manasi Poudel, Dam Prasad Shrestha

**Affiliations:** ^1^ Feline Research Center of National Forestry and Grassland Administration, College of Wildlife and Protected Area Northeast Forestry University Harbin China; ^2^ GoldenGate International College, Tribhuvan University Kathmandu Nepal; ^3^ Environment Protection and Study Center (ENPROSC) Kathmandu Nepal; ^4^ Department of Territory and Agro‐Forestry Systems (TESAF), University of Padua Legnaro PD Italy; ^5^ Paklihawa Campus, Institute of Agriculture and Animal Science (IAAS) Rupandehi Nepal

**Keywords:** carbon stock, forest structure, Nepal, regeneration status, species diversity, sustainable management

## Abstract

Community forestry, a participatory forest management system, encourages forest conservation, enhances carbon sequestration, and advances the Sustainable Development Goals (SDGs) by increasing community ownership of resource management. Community forestry, in terms of policies and practices, directly supports SDG‐1, SDG‐13, and SDG‐15 by promoting community ownership and empowerment as well as ecosystem health. It provides immediate benefits to local livelihoods by enhancing access to ecosystem services such as fuelwood, fodder, and non‐timber forest products. Here, we assess the forest regeneration and conservation value of a community forest in Nepal by recording seedlings, saplings, and mature trees in randomly sampled plots. The plots were structurally complex with rich tree diversity; most plots showed high diameter at breast height (DBH) differentiation and diversity. Most tree species followed an abundance distribution of seedlings > saplings > adults, suggesting “good” regeneration. Tree carbon stock was 137.6 tons per hectare and was positively associated with tree density and diversity and negatively with altitude. An increase of one standard deviation in the diversity index ~0.39 was associated with an 11.7% higher tree carbon stock per hectare. Plot‐level tree height was positively related to diversity at lower tree heights. Overall, community forestry successfully transformed a degraded forest into one of quality regeneration and conservation value within two decades, outperforming the regional average forest carbon stock and diversity. Thus, community forests can act as an effective model for sustainable forest management and are essential tools for policymakers to promote regeneration, structural diversity, carbon storage, tree growth, and sustainable resource utilization.

## 1 Introduction

Forests are among the most productive terrestrial ecosystems (Hattenschwiler et al. 2005; Wang et al. 2005; Bommarco et al. 2013) with key roles in biodiversity conservation, carbon sequestration, and in sustaining rural livelihoods (Peres et al. 2010; Pan et al. 2011; Nitanan et al. 2020). Globally, forests contributed 861 ± 66 Pg of carbon stock, with a net sink rate of 2.4 ± 0.4 Pg per year (Pan et al. 2011), giving themselves a large say in climate change mitigation and greenhouse gas emission reduction planning (Canadell and Raupach 2008; Brockerhoff et al. 2017). Among all available carbon capture and storage technologies, growing trees still remain the most efficient and cost‐effective known method (Bastin et al. 2019). The forest's ability to sequester carbon depends on forest type (Zhou et al. 2013), successional stage (Chazdon et al. 2016), tree species diversity (Ruiz‐Benito et al. 2014), tree density (Rautiainen et al. 2011), forest structure (Forrester 2019), and forest management modalities applied (Norris et al. 2010; Wade et al. 2010), in addition to climatic and geographic factors such as temperature, rainfall, topography, altitude, and edaphic characteristics (Chaturvedi and Raghubanshi 2015; Sharma et al. 2016).

Climate change is a major critical threat for ecosystems, human livelihoods, and global sustainability, requiring immediate and coordinated action across all sectors (Nguyen et al. 2023). Forest conservation is crucial for climate change mitigation, centered on assessing the trade‐offs between forest management practices and their impacts on carbon sequestration and water cycling, along with other co‐benefits such as biodiversity conservation and microclimate regulation (Griscom et al. 2017). Tree species richness, along with healthy regeneration, plays an important role in increasing forest productivity, carbon storage, and resource availability for people (Pretzsch and Schutze 2016; Liu et al. 2018; Zeller et al. 2018). Plant species diversity often boosts net primary production (Karanika et al. 2007) by optimizing light use through functional complementarity, though low‐diversity plantations can also be highly productive depending on species traits (Gough et al. 2019). As a result, multilayered canopies, generally observed in species‐rich forests, sequester more carbon. Another critical variable, forest structure, has a prominent role in many forest processes such as water balance, nutrient cycling, carbon partitioning, and light absorption (Pretzsch et al. 2015). Proposals of using thinning to mitigate short‐term drought (Sohn et al. 2016) and species mixing for reducing long‐term drought susceptibility (Grossiord et al. 2014) highlight the importance of manipulating forest structure and diversity and its mechanism to achieve particular objectives (Saha et al. 2023; Sakib et al. 2024; Ismail et al. 2025). For a forest patch with a given stand density, vegetation growth increases first and then plateaus when the leaf area has peaked (Zeide 2004).

In Nepal, the Chure mountain covers 12.8% of Nepal's land area extending east to west along the north of the Ganges plains (Subedi et al. 2021). The Chure landscape, along with Terai landscape, is a biodiversity hotspot with several types of ecoregions, forests, Ramsar sites, and protected areas (Chaudhary and Subedi 2019); Chure alone has 14 forest ecosystem types (Uprety et al. 2023). On top of these biodiversity values, Chure provides various ecosystem services to locals as well as those on the southern plains (Acharya et al. 2019), especially watershed protection, erosion protection, ground water recharge, and water purification (Bhandari et al. 2016; Bishwokarma et al. 2016; Chaudhary and Subedi 2019). Despite its ecological importance, the region is vulnerable to degradation due to deforestation, erosion, and other anthropogenic pressures (Ghimire et al. 2013; DFRS 2014; PCTMCDB 2017). In recognition of Chure's importance, the Government of Nepal has implemented a 20‐year Master Plan for sustainable conservation (PCTMCDB 2017) and made it a national priority program (NPC 2020). Despite the national effort, Chure forests are disappearing at an annual rate of 0.18% (DFRS 2015). Tree species diversity and regeneration capacity in the Chure region remain lower than that in the midhills and Terai forests of Nepal (DFRS 2015) and stores less carbon (in above ground tree biomass) than the national average: 116.94 t/ha compared to 176.95 t/ha (DFRS 2014, 2015).

Beyond the ecological and environmental variables, the character of forest management practices can determine what kind of forest is grown and protected. Among different forest management practices, community forests are steeped in participatory philosophy, whereby the government hands over a parcel of land to the local community to manage sustainably (Birch et al. 2014). Nepal's experience with community‐based forestry management in the 1970s led to becoming one of the pioneering countries in the early 1990s to devolve substantial aspects of forest management to local communities (Ghimire and Lamichhane 2020). By 2020 there were 22,266 community forest user groups (CFUGs) nationally (DFRS 2020), which covered 41% of national forests and had 2.9 million households as members. This management practice in Nepal led to positive changes in forest conditions (Libois et al. 2022), higher availability of forest products (Adhikari et al. 2007; Pandit and Bevilacqua 2011), lower forest product collection time (Pokharel and Nurse 2004), increased women's participation (Agarwal 2010), higher investment in rural public infrastructure (Paudel 2011), and availability of micro‐credit to vulnerable persons (Dev et al. 2003). In the current REDD+ era, diverse and healthy community‐managed forests with high carbon sequestration and climate change resilience are important goals, as these forests store approximately 21% of the total forest carbon in Nepal (Pokharel and Byrne 2009). Despite differences in the inheritance, the length of conservation—over 30 years of community forestry—gives us an ample timeframe to analyze the quality and structure of regenerated community forests in the ecologically fragile Chure region. In light of the government's conservation effort and Chure region's ecological importance (MOFSC 2016), understanding the status of forests in Chure is a key step in preserving this fragile landscape while simultaneously improving local livelihoods.

To understand the effectiveness of community‐based forestry in the Chure region, we selected a study site that was highly degraded before being transferred to the community in 2001. The study site also had to be near human settlement so that we could get measurable observations of trampling, leaf littering, and fire signs within forest plots. For this, we selected a regenerated community forest in the western Chure region of Nepal. The study site, Tripureshwor Community Forest (TCF), is adjacent to the Birendranagar municipal area, a major city in western Nepal. This study aimed to investigate the carbon stock and biodiversity in the community‐managed forest. We examined the relationships between key ecological and environmental variables of the forest ecosystem, namely DBH, tree height, tree density, species richness, species diversity, elevation, slope, invasive species, and forest carbon stock. To understand ecological mechanisms, the study focused on the following questions:

1. How does tree carbon stock (TCS) vary with key ecological variables such as DBH, tree height, tree density, species richness, species diversity, elevation, slope, and the occurrence of invasive species?
2. What proportion of TCS is constituted by mature trees compared with younger trees across dominant species in the study area?
3. What are the potential impacts of slope, altitude, and the presence of invasive species on carbon sequestration and species diversity in the studied community‐managed forest?

## 2 Materials and Methods

### 2.1 Study Area

**FIGURE 1:**
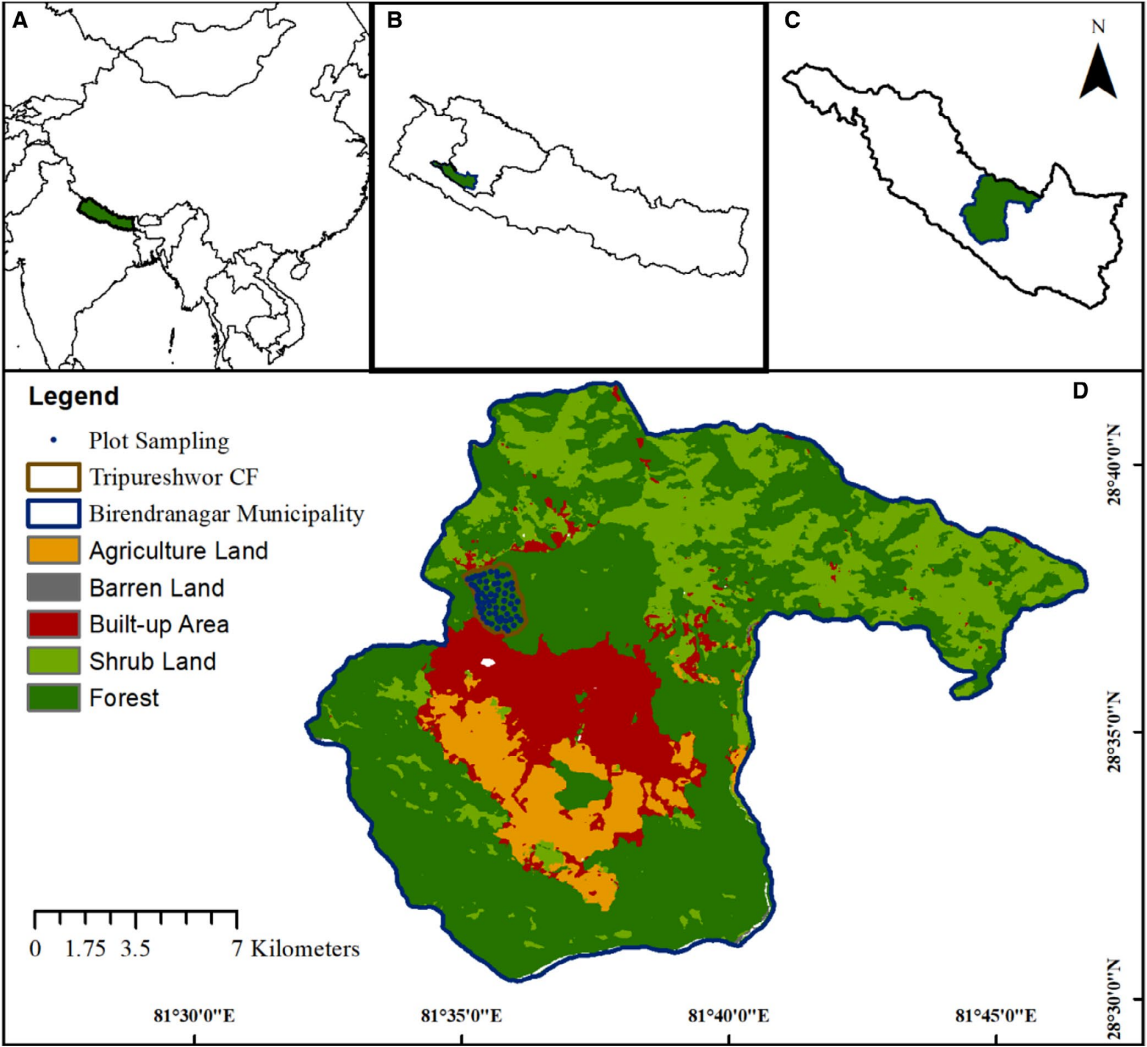
Map of study area showing forest coverage (dark green), agriculture land (orange), settlement area (dark red) and sample plots (blue points). Insets depict the location of the study area regionally (A), within Nepal (B), within Surkhet district (C), and within Birendranagar municipality (D).

Tripureshwor Community Forest (TCF) lies in Birendranagar municipality, in Surkhet district of Karnali Province of Nepal, bounded by 81°35′ to 81°53′ longitude and 28°36′ to 28°41′ latitude (Figure 1). The forest extends from an altitude of 736–1352 m and has terrain with slopes of 15°–80°. It is a dry subtropical forest with three distinct species dominance classes: *Shorea robusta* C.F.Gaertn (broadleaved) dominated forest, mixed forest, and *Pinus roxburghii* Sargent (Coniferous) dominated forest. The study area experiences an average annual precipitation rate of 1609 mm, and the average temperature ranges between 6°C and 36°C (Bhandari 2013). The forest area of 213.95 ha is managed by the Tripureshwor Community Forest User Group (TCFUG) consisting of 830 households. Ethnicity‐wise, Birendranagar municipality's population is grouped into the Khas ethnic cluster (53.8%), followed by the indigenous cluster (18.8%) and Hill Dalits (11.2%). The cooking fuel of choice in the area is firewood (60.1%) and liquefied petroleum gas (36.9%) (Karki 2019).

### 2.2 Data Collection and Analysis

The forest survey was carried out in November 2019 by a team of three researchers and two community forest guards (for a total of 28 working days) based on a simple random sampling method. Altogether, 62 random points were generated using ArcGIS 10.2 (ESRI 2011) within the forest boundaries. Plots were 20 m × 20 m squares, with a minimum of 100 m distance between them. Inside a plot, two 5 m^2^ in opposite corners were used to sample saplings, and another four 1 m squares in each corner were allocated for counting seedlings (Figure S1).

Additionally, GPS location, altitude, slope, fire sign, trampling, leaf litter condition, and presence of invasive herbaceous species were recorded for all plots. The fire sign variable was dichotomous, recording any evidence of fire in the forest plot. Invasive species presence, trampling extent, and leaf littering were based on visual observation. Invasive species presence was noted by checking plant species in the plot using an invasive species checklist (Shrestha 2016). Trampling and leaf littering were recorded as one of three levels: (a) low, (b) medium, and (c) high. Specifically, if the sampled plot had less than 33% of its area trampled or covered by leaf litter, then it was classified as a low trampled or low leaf litter covered plot following Maharjan et al. (2006). Similarly, 66% of the area was the cutoff between medium and high values. Mathematical slope correction was applied to plots during the analysis following Abella et al. (2004).

A field manual of forest resource assessment (FRTC 2019) was used to classify vegetation as trees, saplings, and seedlings. Tree height and DBH (1.3 m from ground level) were measured for individual trees inside the sample plots using a Silva Clinometer (Silva‐Clino Masters, Sweden) and DBH tape, respectively, following Aryal et al. (2022). Trees were categorized as woody plant species with multiple secondary branches visible above ground on a single main stem or trunk, distinct apical dominance, a DBH ≥ 5 cm, and a height ≥ 1.5 m following Tang et al. (2023). Plants with a DBH less than 5 cm and a height greater than 1.3 m were categorized as saplings; whereas, plants less than 1.3 m in height were classified as seedlings, following Wassie et al. (2010). All tree (DBH ≥ 5 cm) and shrub species (i.e., woody plants with multiple stems, usually shorter than trees) were identified in the field following Shrestha et al. (2018). For each sampling plot, we calculated the coefficient of variation (CV = SD/mean) of DBH to evaluate DBH differentiation and classified CV following Tíscar and Linares (2011) as: CV < 0.05: very low differentiation; 0.05 < CV < 0.15: low differentiation; 0.15 < CV < 0.30: moderate differentiation; 0.30 < CV < 0.60: high differentiation; and CV > 0.60: very high differentiation.

Plant species (tree) data were analyzed for species richness, density, frequency of occurrence, Shannon–Wiener index (H′) (Shannon and Weaver 1949), Pielou's evenness (J) (Pielou 1966), basal area, canopy cover, volume, above ground tree biomass (AGTB), and carbon stock contributed by trees in the study area following Chave et al. (2005) and national forest resource assessment of Chure forests (DFRS 2014). The regeneration status of species was determined based on the number of trees, saplings, and seedlings (Khan et al. 1987; Malik and Bhatt 2016) as follows: “good” if seedlings > saplings > adults; “fair” if seedlings > or ≤ saplings ≤ adults; “poor” if no seedlings but presence of saplings (saplings may be <, > or = adults); “no” if only adults are present; and “new” if no adults but only seedlings or saplings.

During the survey, the survey team leader entered observations into a plot‐wise data collection form, which was then coded and parsed using MS Excel and later analyzed in R 4.2.2 (R CoreTeam 2024) using the fixest package (Berge 2018). We employed multiple linear regression with plot‐wise TCS as the dependent variable, tree density and tree species richness as key explanatory variables, with slope, altitude, slope‐corrected plot area, trampling, leaf littering, average tree DBH, and average tree height as control variables (i.e., covariates). Before constructing the model, the auto‐correlation of the explanatory variables was tested to verify their independence. We added individual terms, interaction terms, quadratic terms, and interaction between linear and quadratic terms of average tree DBH and average tree height. The most parsimonious models with the fewest average tree DBH and average tree height‐related terms were selected using *R* ^2^, *R* ^2^ adj., RMSE, and the statistical significance of the variables within the models (Burnham et al. 2011); the three most parsimonious models are presented in the results. Robust standard errors (HC5) (Cribari‐Neto et al. 2008), calculated using the sandwich package (Zeileis 2004), are reported in the results to account for possible heterogeneity. We also modeled the relationship between the Shannon–Wiener diversity index and plot‐wise characteristics with multiple linear regression.

## 3 Results

### 3.1 Forest Composition and Structure

Most of the sample plots had a south‐west aspect (88.7%), and the slope of terrain ranged from 15° to 80°. The altitudinal variation was between 736 and 1352 m above mean sea level (msl). In the study area, 91 plant species (spp.) were identified (71 tree spp., 16 shrubs spp. and 4 climbers spp.); *Shorea robusta* C.F.Gaertn was the dominant tree species (28.9%) followed by *Pinus roxburghii Sargent* (9%). Although the top seven dominant tree spp. represented 87.5% of the total basal area, they only made up 68.4% of the total tree volume (Table 1). Average density was 1461 trees ha^−1^, average basal area was 75.2 m^2^ ha^−1^, and average volume was 324.9 m^3^ ha^−1^. The tree density value ranged from 422.6 trees ha^−1^ ( *Shorea robusta* C.F.Gaertn) to 0.6 trees ha^−1^ ( *Tamarindus indica* L.) (Table 1). Out of 62 plots, 72.6% of plots showed very high DBH differentiation (CV > 0.6), and 25.8% of the plots showed high DBH differentiation (0.3 < CV ≤ 0.6), while only one plot showed moderate DBH differentiation (0.15 < CV ≤ 0.3).

**TABLE 1:** The seven major tree species recorded across all plots, and their respective carbon contribution.

Species	% of total trees across all plots	Density (trees ha^−1^)	Presence (%) among all plots			TCS (t ha^−1^)
			Tree	Sapling	Seedling	
*Shorea robusta*	28.9	422.6	85.5	83.9	48.4	100.7
*Syzygium cumini*	9.0	131.9	83.9	72.6	93.5	3.8
*Mallotus philippensis*	8.6	126.3	82.3	40.3	53.2	2.7
*Catunaregam spinosa*	5.1	74.6	59.7	30.6	22.6	0.8
*Trichilia connaroides*	4.6	67.9	50.0	43.5	51.6	1.6
*Holoptelea integrifolia*	4.5	65.7	80.6	25.8	25.8	0.7
*Pinus roxburghii Sargent*	4.4	64.5	14.5	1.6	1.6	15.0

Abbreviation: TCS, tree carbon stock.

The average shrub density was 57.7 ind. ha^−1^ (individuals per hectare). Average plot‐wise tree DBH ranged from 12.4 cm to 29.0 cm. Similarly, the species richness, volume, and tree density varied widely among plots (Table 2). The tree diameter (DBH) distribution showed a classical inverted J‐curve (Figure S1), except for *Pinus roxburghii Sargent* (Figure S2).

**TABLE 2:** Plot‐wise summary of structural, geographical, and species richness variables.

Parameters	Mean	SD	Minimum	Maximum
Tree carbon stock (t ha^−1^)	137.6	148.4	23	864.8
Basal area (m^2^ ha^−1^)	75.2	68.6	16.2	367.9
Volume (m^3^ ha^−1^)	325.0	392.2	38.1	2088.8
Species richness (species plot^−1^)	12.0	3.0	6.0	19.0
Shannon–Wiener diversity index (H′)	2.0	0.4	0.9	2.7
Evenness (E)	0.8	0.1	0.5	1.0
Density (trees plot^−1^)	44.3	17.7	18.0	111.0
Average DBH (cm)	18.4	4.0	12.4	29.0
Slope (°)	34.3	19.5	15.0	80.0

### 3.2 Forest Diversity, Regeneration and Carbon Stock

Overall, saplings of 61 spp. and seedlings of 56 spp. were identified in the study forest. *Shorea robusta* C.F.Gaertn had the highest sapling frequency (83.9%) (Table 1), whereas saplings of 12 species were present in single plots only. Among seedlings, *Syzygium cumini* (L.) Skeels was the most dominant in terms of frequency, being present in 58 plots out of 62. We did not find seedlings for 21 tree spp., and for 9 spp., seedlings were present only in individual plots. Among tree spp., the total sapling and seedling densities were 6842 and 86,764 ind. ha^−1^, respectively. The Shannon–Wiener diversity index (H′) and evenness (E) for tree species, across all the plots, were 2.9 and 0.7, respectively. Regeneration‐wise, most of the species (52.1%) showed good regeneration while the rest had fair regeneration (12.7%), poor regeneration (9.9%), no regeneration (19.7%), and new regeneration (5.6%).

Carbon stock differed substantially between the plots from 23 to 864.8 t ha^−1^ (Table 2). The biggest contributors, *Shorea robusta* C.F.Gaertn (214.2 t ha^−1^), *Pinus roxburghii Sargent* (31.9 t ha^−1^), and *Syzygium cumini* (L.) Skeels (8.2 t ha^−1^), accounted for 86.8% of the total biomass.

The TCS was found to be positively associated with tree density (*p* < 0.01) and the Shannon–Wiener diversity index (*p* < 0.05), but negatively associated with altitude (*p* < 0.05). On average, an increase of one standard deviation in the diversity index ~0.39 is related to an increase of 100 × ((0.248 × 0.39) − 1) ≈ 11.7% in TCS. The slope of the plot is significant in the model. Adding interaction terms of DBH and heigh, and DBHsquared, improves the model's predictive power (*R* ^2^) and significance of diversity's impact on TCS (Table 3).

**TABLE 3:** OLS model of tree carbon stock and plot characteristics.

Dependent variable		Log (Tree carbon stock t ha^−1^)		
Model		(1)	(2)	(3)
Variables				
Slope (degree)		0.020*** (0.003)	0.020*** (0.003)	0.018*** (0.003)
Altitude (m)		−0.001 (0.0005)	−0.001* (0.0005)	−0.001** (0.0004)
Shannon–Wiener diversity index (H′)		0.193* (0.117)	0.151 (0.115)	0.284** (0.114)
Tree density		0.017*** (0.005)	0.019*** (0.004)	0.018*** (0.004)
Trampling (b.c: Low)	Medium	0.060 (0.126)	0.048 (0.120)	0.105 (0.111)
	High	0.056 (0.178)	0.034 (0.176)	0.070 (0.161)
Litter Condition (b.c: Low)	Medium	0.258 (0.201)	0.289* (0.170)	0.284* (0.152)
	High	0.173 (0.281)	0.291 (0.267)	0.287 (0.274)
Average tree DBH		0.186*** (0.019)	0.317*** (0.111)	0.553*** (0.102)
Average tree height		−0.136* (0.074)	0.340 (0.365)	−0.614 (0.451)
Average tree DBH × Average tree height			−0.025 (0.019)	0.023 (0.024)
Square of average tree DBH				−0.012*** (0.004)
(Intercept)		0.572 (0.702)	−1.853 (2.114)	−1.691 (1.740)
Fit statistics				
Observations		62	62	62
*R* ^2^		0.781	0.789	0.822
*R* ^2^ adj.		0.738	0.742	0.778
RMSE		0.38	0.37	0.34

*Note:* Robust standard errors (HC5) in parentheses.

Abbreviations: b.c, base category; RMSE, root mean square error.

* *p* < 0.1.

** *p* < 0.05.

*** *p* < 0.01.

The plot‐level Shannon–Wiener diversity index was not significantly related with plot altitude. However, diversity was significantly correlated with sapling abundance (*p* < 0.05), slope (*p* < 0.05), and tree height (*p* < 0.05) (Table 4). The average marginal effect of plot‐level tree height on diversity showed positive relationship at lower tree heights, whereas negative relationship at higher tree heights (Figure S2), indicating a nuanced relationship between tree height and diversity across plots.

**TABLE 4:** OLS model of tree Shannon–Wiener diversity index (H′).

Dependent variable	Shannon–Wiener diversity index (H′)		
Model	(1)	(2)	(3)
Variables			
Number of seedling	0.001 (0.004)	−0.001 (0.004)	0.000 (0.004)
Number of sapling	0.015*** (0.006)	0.012** (0.005)	0.078** (0.037)
Altitude (m)	−0.004 (0.005)	−0.006* (0.004)	−0.006 (0.004)
Square of altitude	2.11e^−6^ (2.12e^−6^)	3.04e^−6^ * (1.74e^−6^)	2.83e^−6^ (1.76e^−6^)
Slope (degree)	0.006** (0.003)	0.006* (0.003)	0.006** (0.003)
Average tree height	−0.130* (0.077)	1.364** (0.658)	1.302** (0.576)
Square of average tree height		−0.135** (0.056)	−0.097** (0.046)
Number of sapling × average tree height			−0.012* (0.007)
(Intercept)	3.871 (2.364)	1.108 (2.508)	0.109 (2.533)
Fit statistics			
Observations	62	62	62
*R* ^2^	0.196	0.319	0.363
*R* ^2^ adj.	0.108	0.230	0.267
RMSE	0.34	0.32	0.31
Estimated turning point	—	5.051	4.839

*Note:* Robust standard errors (HC5) in parentheses.

Abbreviation: RMSE, root mean square error.

* *p* < 0.1.

** *p* < 0.05.

*** *p* < 0.01.

### 3.3 Trampling, Leaf Littering and Invasive Species

The trampling and leaf litter conditions were generally medium (61.29% and 77.19%, respectively); whereas, fire signs in plots were low (6.45%). All plots had invasive species. Major invasive species in the forest were *Ageratum conyzoides* , *Ageratum houstonianum* , *Bidens pilosa* , *Lantana camara* , *Parthenium hysterophorus* , and *Xanthium strumarium* . During the field survey, it was noted that in plots with a higher density of invasive species, seedling density was lower.

## 4 Discussion

The Forest Regulations 2022 of Nepal (MOFE 2022) enable community forestry groups, in consultation with the divisional forest office, to implement forest management plans of their choice under the constraints according to sustainable harvesting rates. Further, harvested timber is sold in accordance with a predefined priority, i.e., members are preferred over the district forest product supply committee and outsiders. Community forest user group members are sold timber at a nominal fee for their personal use, and it is illegal for them to arbitrage timber in open markets (Sunam and McCarthy 2010). This focus of regulation on sustainability and meeting local needs, and TCF's dominantly conservative management plan over the years, selecting dead, dying, deformed, and over‐mature trees for local usage means that the study area had ample time to regenerate from its degraded state at the time of acquisition by the local community, which is reflected by the inverted J‐shaped DBH distribution curve. Similar inverted J‐shaped relationships in several community forests in different parts of Nepal have been reported by Ayer et al. (2022), Pandey and Pokhrel (2021), Poudel and Devkota (2021), Suwal et al. (2015), and Shrestha (2005). Our results of a non‐J‐shaped curve are consistent with prior studies (Sharma et al. 2020; Pandey and Pokhrel 2021).

Large older trees are a valuable resource for biodiversity (Franklin et al. 2002). For instance, veteran individuals of *Shorea robusta* , *Syzygium cumini* (L.) and *Pinus roxburghii* show a distinctive architecture that supports a higher diversity of plants and animals with varied types of habitat different from those offered by younger trees (Bhattarai et al. 2021). Since there were no reports of major forest fires and other natural disasters in the last decade, we found some stands with large trees (DBH > 200 cm) of *Shorea robusta* . This result is in contrast with Acharya (2004), who found no such DBH distribution due to burnings, and Ghimire and Lekhak (2007), who reported the absence of *Abies spectabilis* (D. Don) Mirb with larger diameter (DBH > 45 cm) due to locals' harvesting. The presence of stands with large trees suggests that TCF has managed to protect them from illegal logging, considering the high economic valuation of wide *Shorea robusta* boles. In fact, most of the carbon in the forest was stored by *Shorea robusta* , since mature trees of other species were less abundant. But overall, the CV of DBH was generally in the very high category, which is a sign of a complex forest structure. This structure, plus the normal inverted J‐shaped DBH‐tree frequency curve, indicated the stable population with good potential for regeneration and recruitment, as the successful regeneration of a given forest requires the occurrence of a sufficient number of young trees, saplings, and seedlings within the population (Hanief et al. 2016). The density of trees (ha^−1^) too reflects good regeneration. The soils of the Chure region, formed from limestone, sandstone, and parent materials, are slightly basic and rich in organic matter, which supports the growth of seedlings and saplings (Puget and Lal 2005).

In the last decade, TCS has been increasing in the Chure region (Subedi et al. 2022); we also report higher tree carbon stock (TCS) (137.6 t ha^−1^) compared to the average national value (84.73 t ha^−1^) for the Chure region, which includes community forest and national forest (DFRS 2014). Direct comparison with other study sites in the Chure region will often be biased as there are several important factors that affect TCS, e.g., slope (Bohara et al. 2021), aspect (Mandal et al. 2017), species diversity (Aryal et al. 2018), dominant species (Pandey et al. 2014), invasion (Ulak et al. 2016), altitude and geographical factors (Baral et al. 2009), climatic factors (KC et al. 2013), and edaphic factors (Wang et al. 2001).

Another important component while comparing TCS across studies is methodological. Our TCS result is corrected for slope, and as a result, it is higher than if left without correction. Despite these factors, a simple TCS comparison across other results in western Chure puts our TCS result at a higher end of the spectrum of reported TCS (DFRS 2014; Bhusal and Bhattarai 2020; Charmakar et al. 2021; Lamichhane et al. 2021; Subedi et al. 2022). Our species diversity (H′ = 2.91) is also higher than the national average (1.93) for the Chure region, including community and national forests (DFRS 2014). This higher diversity refutes the concern that CFUG's potential focus on yielding economically valued timber species has led to lower species diversity.

In the literature, species richness is hypothesized to alter the rate of carbon sequestration through complementarity effects; individuals in species‐rich communities optimize resources by niche partitioning, leading to higher carbon storage (Cardinale et al. 2009; Díaz et al. 2009). The general consensus is the existence of a positive relationship between plant species richness and productivity (Hector et al. 1999; Huang et al. 2018), through efficient use of soil nutrients and sunlight, leading to increased carbon sequestration. We find a statistically significant and positive relationship between tree carbon storage and species richness, similar to earlier results (Pragasan 2020; Charmakar et al. 2021; Joshi et al. 2021). The altitudinal variation was negatively related to carbon storage, which, too, is supported by the literature (Zhu et al. 2010; Nath et al. 2021).

We also found a positive relationship between tree density and carbon stock, when we control for average plot tree DBH, average plot tree height, and their interaction terms. This result corroborates earlier positive relation of tree density with carbon stock (Pragasan 2014; Behera et al. 2017). In contrast with earlier studies (Aryal et al. 2018), we did not find decline of species richness with altitude. Rather, tree height and number of saplings were the strongest predictors of plot‐wise species richness. This result is similar to Pandey et al. (2014) who found that forests were moving toward fewer species as the tree matured. The total marginal effect of average tree height on species diversity supports this observation; at lower heights, the relationship was positive, and vice versa. A possible explanation might be that as the average tree height gets larger, the crown cover of the faster growing larger trees provide competition against the slow growing species. The relationship between canopy width and tree height is, generally, linear and stable (Arzai and Aliyu 2010), mainly because of natural selection pressure forced by photosynthesis in favor of maximum light interception (Anten 2005). With increased height, trees' metabolic and growth requirements increase and trees are more likely to evolve to have wider canopies in order to maximize light interception and increase their photosynthetic rates (Jahnke and Lawrence 1965). Thus, in regenerating forests, plots with lower average tree height are likely to have more species since competitive species are yet to dominate less competitive species and drive them off from the plot. The importance of slope in the species richness regression model is also likely derived from the photosynthetic factor as plants having lower heights are more likely to receive sunlight in sloped plots vis‐à‐vis flat plots resulting in more plot‐wise species. Further, an inverted J‐curve characteristic of our study area, i.e., dominant density of trees with low crown cover, supports sunlight reaching to the ground level helping photosynthesis mechanism of understory vegetation such as saplings, seedlings, and shrubs, allowing the forest to maintain higher vegetation diversity and density.

In all forest plots, we found the presence of invasive species. Similar widespread presence has been reported in the Terai region of Nepal (Gaudel et al. 2016; Chaudhary et al. 2020), central midhills (Dyola et al. 2020), and Chitwan –Annapurna Landscape (Shrestha et al. 2019). While we did not do a quantitative analysis relating to invasive species, our observations indicated that plots with a higher abundance of invasive species tended to have lower seedling density, in line with Gaudel et al. (2016), Chaudhary et al. (2020) and Dyola et al. (2020). Since we only collected presence –absence data, the collected data had no variation by virtue of invasive species being present in all plots, so we could not conduct empirical tests to validate our observational results. A better strategy would have been to collect plot‐ and species‐wise invasive species density, which could then be used to understand the effect of invasion on carbon storage, species diversity, and the distribution of invasive species with plot characteristics. Similarly, leaf littering and trampling were present in about two‐thirds of the plots. The data generation of both of these variables were by researchers' observations and, as a result, they lack scientific rigor and variation; thus, they were not influential in our models as we had originally envisioned.

Furthermore, future research should expand on our findings to explore how community‐managed forests enhance biodiversity and carbon sequestration capacity under the cumulative impacts of climate change and anthropogenic disturbance. Since it has been identified that large trees have significant roles in carbon sequestration (Stephenson et al. 2014), it is crucial to identify appropriate strategies to protect mature tree individuals or stands in regions with high deforestation and degradation. Future studies should concentrate on identifying the long‐term effects of selective tree thinning and management strategies used to control invasive species on the dynamics of forest structure, carbon sequestration capacity, and the influence on the resilience and productivity capacity of forests. The identification of advanced modern technologies for long‐term ecological monitoring is essential for tracking forest changes and refining management practices. Incorporating socio‐economic research on community engagement in forest governance will serve to ensure beneficial and sustainable conservation plans, driving community‐based forest management models worldwide and supporting the mitigation of climate change, preservation of biodiversity, and sustainable development.

## 5 Conclusion

Our analyses of forest measurements relevant to forest regeneration and carbon storage in a site in western Chure Hills, Nepal, around two decades after it was handed over—in a degraded state—to a community for community management in 2001 suggest a number of main findings. First, the claims that the TCFUG's interest in growing high‐valued timber species to the detriment of forest species richness appear to be unfounded; in fact, species diversity in the studied community forest was higher than the average for the Chure region. Second, forest structure in most cases followed seedlings>saplings > adult trees by number of individuals per species, typically regarded as favorable signs of forest regeneration. Tree carbon stock (TCS) highly varied across plots, and the aggregate value was higher than the average for the region. Invasive species were present in all plots. Hence, we recommend going beyond the presence/absence of invasive species during future research in the region. Overall, the results of forest composition, structure, diversity, and carbon stock suggest that this community‐managed forest has good regeneration characteristics and demonstrates the importance of community‐managed forests more broadly as an important policy tool for promoting forest restoration and carbon storage. Overall, in this study, community forestry is found to enhance resilience to climate change by restoring degraded forests, supporting biodiversity, and promoting carbon storage, aligning with multiple Sustainable Development Goals (SDGs). These findings affirm that community‐managed forests could be a vital policy tool for advancing forest restoration, climate resilience, and sustainable development at local to regional scales.

## Ethics Statement

TCFUG reviewed the research proposal and provided permission for the fieldwork inside the community forest.

## Conflicts of Interest

The authors declare no conflicts of interest.

## Supporting information


Figure S1.
Figure S2.

## Data Availability

Data are available on request from the authors.
