# Supplementary material for: Forest Structure, Diversity, and Regeneration in a Community‐Managed Forest of Nepal: A Model for Carbon Sequestration and Sustainable Management
Source: Plant Environ Interact. 2025 Mar 16;6(2):e70044. doi: 10.1002/pei3.70044 (PMC11910969; doi:10.1002/pei3.70044)
Supplement: Supplementary file 1 — Figure S1. Figure S2. [file PEI3-6-e70044-s001.docx]

# Supplementary Figures and captions


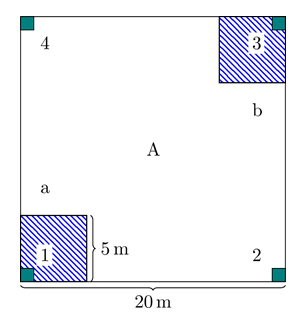


SI Figure 1: Sample plot scheme. Square plot of 20m length (A in figure) was used for the study. In two opposite corners of the square plot, squares of 5m length (a and b in figure) were used for saplings. Further 1 m square plots in each corner of the square plot (1, 2, 3, and 4 in figure) were utilized for seedling counting.


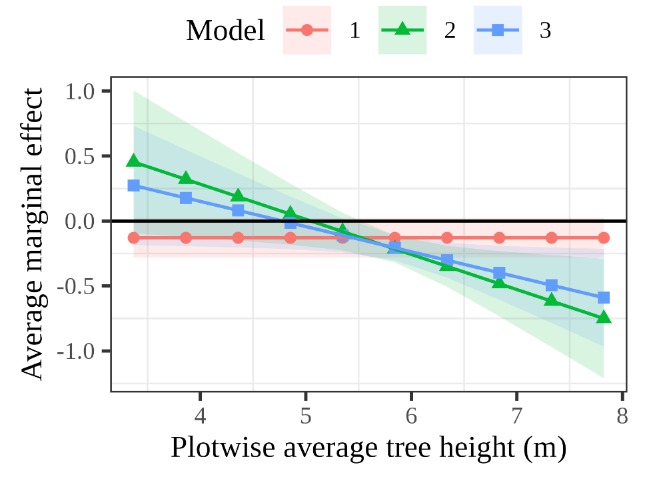


SI Figure 2: Average marginal effect of plot-wise average tree height on tree Shannon-Wiener diversity index (H').


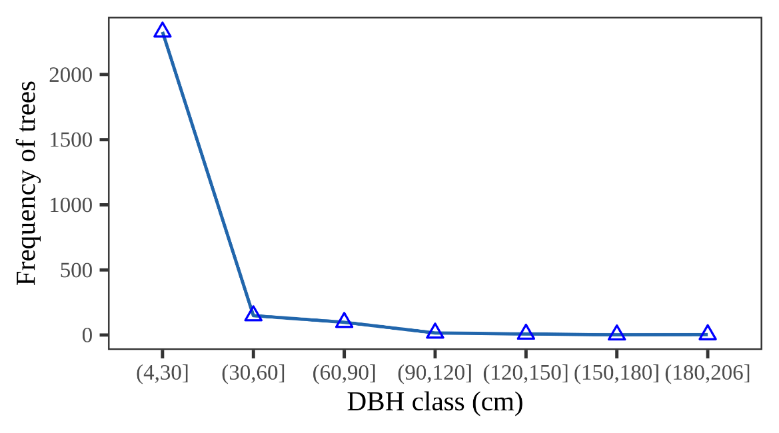


SI Figure 3: DBH size class distribution of tree species. The number of trees and DBH are inversely related.


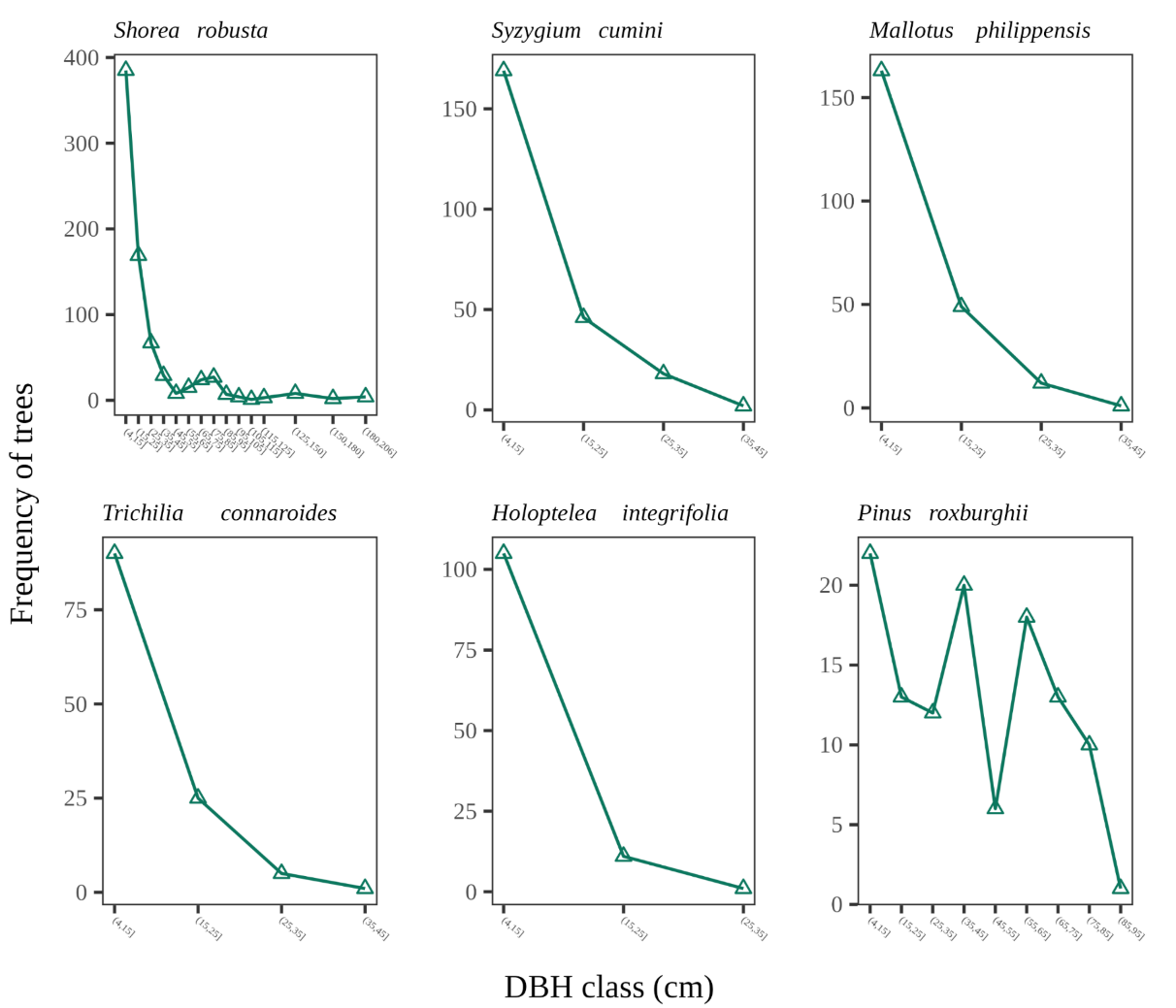


SI Figure 4: DBH size class distribution of individuals tree species. The number of trees and DBH are inversely related, except Pinus roxburghii Sargent.
